# Assessing *Aspergillus oryzae* as a Fungal Chassis for Cyanobacterial Biosynthetic Gene Clusters via Heterologous Expression of the Lyngbyatoxin Biosynthetic Pathway

**DOI:** 10.3390/jof12070481

**Published:** 2026-07-01

**Authors:** Sameera Jayasundara, Tahir Ali, Bisola Adeyemi, Bagyashri Krishnamoorthy, Calvin A. Henard, Kent D. Chapman, Elizabeth Skellam

**Affiliations:** 1 Department of Chemistry, University of North Texas, 1155 Union Circle, Denton, TX 76203, USA; sameerajayasundara@my.unt.edu (S.J.); tahir.ali@unt.edu (T.A.); bisolaadeyemi@my.unt.edu (B.A.); bagyashrikrishnamoorthy@my.unt.edu (B.K.); 2 BioDiscovery Institute, University of North Texas, 1155 Union Circle, Denton, TX 76203, USA; calvin.henard@unt.edu (C.A.H.); kent.chapman@unt.edu (K.D.C.); 3 Department of Biological Sciences, University of North Texas, 1155 Union Circle, Denton, TX 76203, USA; 4 Advanced Materials and Manufacturing Processes Institute, University of North Texas, 1155 Union Circle, Denton, TX 76203, USA

**Keywords:** *Aspergillus oryzae*, biotransformation, codon optimization, cyanobacterial natural products, fungal chassis, heterologous expression, lyngbyatoxin A

## Abstract

Cyanobacterial biosynthetic gene clusters (BGCs) remain difficult to reconstruct in heterologous systems, yet the suitability of fungal hosts for expression of cyanobacterial pathways remains largely unexplored. Here, the well-characterized lyngbyatoxin A (LTXA) biosynthetic gene cluster was used as a model system to evaluate the capacity of *Aspergillus oryzae* to support cyanobacterial NRPS biosynthesis. The lyngbyatoxin biosynthetic genes (*ltxA*, *ltxB*, and *ltxC*) were individually cloned and expressed in *Aspergillus oryzae* NSAR1 under the control of an inducible promoter. Metabolite production was assessed by LCMS, while transcriptional analysis was performed using RT-PCR. Codon-optimized constructs and precursor feeding experiments were used to evaluate pathway functionality. No detectable production of LTXA or pathway intermediates was observed following co-expression of *ltxA–C* despite confirmed transcription of *ltxB* and *ltxC*. RT-PCR analysis could not reliably detect *ltxA* transcripts for unknown reasons. In contrast, expression of a codon-optimized *ltxC* enabled biotransformation of indolactam V to LTXA in *A. oryzae*, confirming functional expression of the prenyltransferase. Overall, this work establishes lyngbyatoxin biosynthesis as a useful model system for evaluating fungal chassis compatibility with cyanobacterial BGCs and provides insights for future engineering of fungal platforms for natural product biosynthesis.

## 1. Introduction

**Figure 1:**
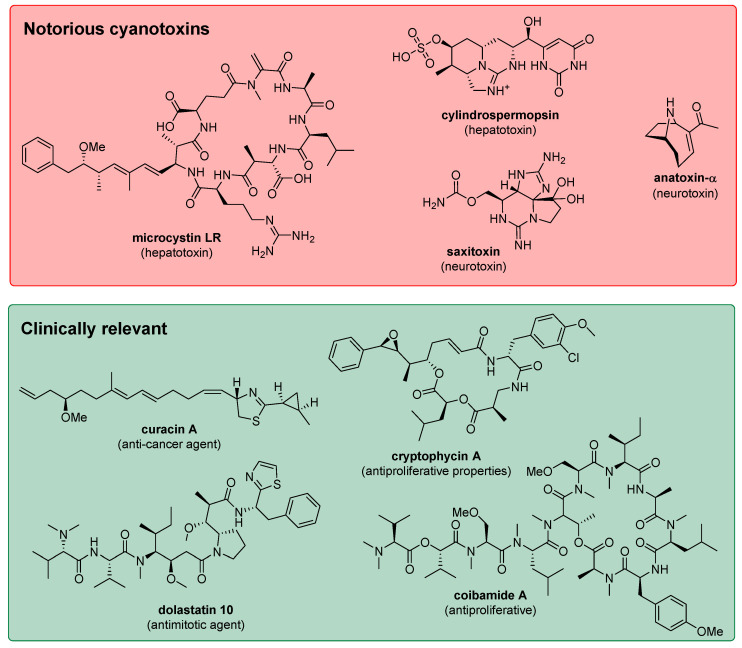
Examples of cyanotoxins and clinically relevant natural products of cyanobacterial origin. Microcystin LR is produced by *Microcystis aeruginosa*; cylindrospermopsin is produced by *Cylindrospermopsis raciborskii*; saxitoxin is produced by *Aphanizomenon*; anatoxin-α is produced by *Anabaena* sp.; curacin A is produced by *Lyngbya majuscula*; cryptophycin A is produced *by Nostoc* sp., dolastatin 10 is produced by *Caldora penicillata*; coibamide A is produced by *Leptolyngbya* sp.

Cyanobacterial natural products represent a diverse source of bioactive molecules with pharmaceutical and biotechnological potential (Figure 1) [1,2,3]. However, access to these compounds is often limited by the slow growth of native producers and the lack of robust genetic tools [4], making heterologous expression an attractive strategy for their production and study [5,6]. Despite advances in pathway engineering, heterologous expression of cyanobacterial biosynthetic gene clusters remains challenging [7,8], particularly for large multi-domain non-ribosomal peptide synthetase (NRPS) systems. Differences in codon usage, transcriptional regulation, and protein folding between hosts can hinder functional expression, and successful reconstitution of complete pathways is rare [4,9,10,11].

**Scheme 1:**
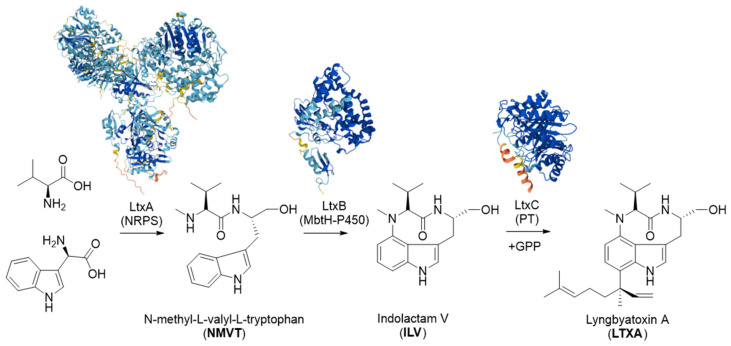
Biosynthesis of LTXA. The NRPS gene *ltxA* encodes the following domain organization A-NM-T-C-A-T-R, where A = adenylation, NM = N-methyltransferase, T = thiolation, C = condensation, R = reductase. *ltxB* encodes LtxB, a P450 fused with an MbtH-like protein; *ltxC* encodes LtxC, a prenyltransferase (PT).

Fungal hosts such as *Aspergillus oryzae* are attractive alternatives for heterologous expression due to their genetic tractability and established use in natural product biosynthesis [12,13,14], yet their suitability for expression of cyanobacterial pathways remains largely unexplored. In this study, we investigated the heterologous expression of one of the best-characterized cyanobacterial NRPS systems, lyngbyatoxin A (LTXA), a protein kinase C (PKC) activator, which has previously been reconstructed in several prokaryotic hosts and therefore serves as an ideal benchmark pathway for evaluating fungal chassis compatibility. By using a genetically and biochemically tractable pathway with known intermediates, precursor requirements, and tailoring reactions (Scheme 1) [15], failures in metabolite production can be interpreted mechanistically and used to identify barriers to pathway portability across kingdoms. Importantly, the required valine, tryptophan, S-adenosyl-methionine, and geranyl pyrophosphate (GPP) precursors are readily available in *A. oryzae* NSAR1; the GC content of the *ltx* genes (~45%) is similar to that of *Aspergillus* genes (~52%), and the expression system enables independent cloning of each gene under the control of a strong inducible fungal promoter [12]. Our results reveal technical challenges with expressing *ltxA* in a fungal host while demonstrating that downstream tailoring enzymes can remain functionally active in *Aspergillus* sp. if codon-optimized.

## 2. Results

### 2.1. Reconstruction of the Lyngbyatoxin BGC in A. oryzae

As PKC is a crucial signaling enzyme in fungi and a potential target for antifungal drugs [16,17], the sensitivity of wild-type *A. oryzae* NSAR1 was established by cultivating in the presence of up to 200 μg LTXA in a 100 mL DPY media flask. Over the seven-day observation period, *A. oryzae* NSAR1 grew normally, and organic extracts of the mycelium and culture broth confirmed that LTXA was not modified by native enzymes. Based on three replicate extractions, over 90% of the added LTXA sample could be recovered from cell samples, whereas less than 5% could be recovered from the culture broth (Table S10). These experiments established that *A. oryzae* NSAR1 could tolerate exogenous LTXA and no evidence of metabolic modification was observed under our assay conditions.

The three essential *ltx* genes were commercially synthesized and individually cloned under the control of the inducible *amyB* promoter in the pTYGS series of expression plasmids [12,18]. As NRPS activation requires phosphopantetheinylation, both the broadly utilized *Bacillus subtilis sfp* phosphopantetheinyl transferase (PPtase) and the cyanobacterial *hetI* PPtase from *Nostoc* sp. PCC 7120 [19] were evaluated to maximize the likelihood of functional pathway activation in the fungal host. The *ltxA*, *ltxB*, and *ltxC* expression plasmids were confirmed by sequencing and co-transformed into *Aspergillus oryzae* NSAR1 via PEG-mediated protoplast transformation together with a fourth plasmid encoding either *sfp* or *hetI.* Resulting transformants were subjected to three rounds of selection on Czapek–Dox medium lacking arginine, adenine, methionine, and ammonium sulfate before transfer to liquid DPY medium for cultivation. After seven days, mycelia were separated from the culture broth, and both fractions were extracted twice with EtOAc prior to concentration and LCMS analysis.

LCMS analysis revealed no detectable production of LTXA, known lyngbyatoxin analogues [20], or identifiable pathway intermediates in any co-expression strain despite confirmed transcription of *ltxB* and *ltxC.* Transcripts were verified by sequencing the PCR products to confirm that they are free of mutations. RT-PCR on mRNA collected from transformants cultivated for 3–4 days determined that *ltxA* never produced full-length transcripts, despite being fully integrated into the genome; when transcripts could be detected, they were truncated (Figure S6). We do not know why *ltxA* failed to produce full-length transcripts, yet all other genes did. Since the *amyB* promoter is inducible by starch or starch-derived oligosaccharides and repressed by glucose, we adjusted our cultivation parameters in an attempt to modify transcription rate and timing. However, cultivation parameters alone were insufficient to induce production of LTXA (Table 1).

### 2.2. Biotransformation of Indolactam V by LtxC in A. oryzae

**Figure 2:**
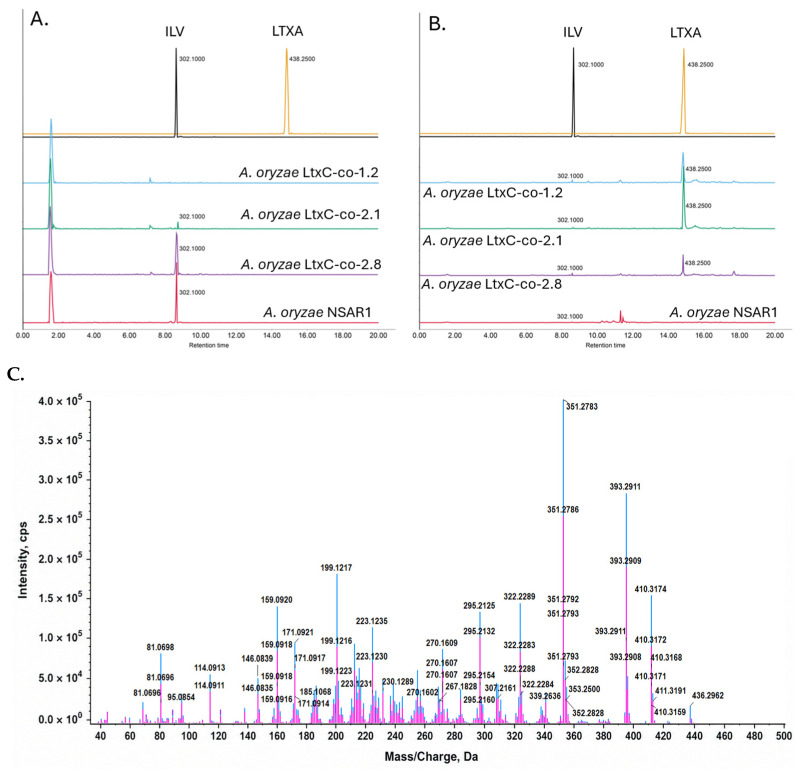
LCMS analysis of *A. oryzae* + *ltxC*-co transformant extracts after supplementation of ILV: (**A**) media extract showing trace amounts of ILV but no LTXA; (**B**) cell extracts showing production of LTXA and no ILV; (**C**) MS/MS overlay of LTXA standard (blue) and the organic extract from a transformant (pink).

Since cyanobacterial gene sequences are known to utilize rare codons [11,21], *ltxC* was re-synthesized codon-optimized (*ltxC*-co) for *Aspergillus* sp. and transformed into *A. oryzae* NSAR1. In parallel experiments, 100 μg of ILV was added to individual *A. oryzae* + *ltxABC*/*ltxC*-co transformants in liquid DPY medium and incubated at 28 °C, shaking at 110 RPM. After seven days, the cultures were extracted and analyzed by LCMS. While ILV (*m/z* 302.1863 *=* [M+H]^+^) could be detected in the *A. oryzae* + *ltxC*-co media extract (Figure 2A), less was detected in the cell extracts (Figure 2B). Although LTXA (*m/z* 438.3115 = [M+H]^+^) was not detected in the media extracts of *A. oryzae* + *ltxC*-co (Figure 2A), it could clearly be detected in the cell extracts (Figure 2B). The production of LTXA in *A. oryzae* + *ltxC*-co transformants was confirmed by MS/MS analysis with a commercial standard (Figure 2C). In contrast, no conversion of ILV to LTXA was detected in *A. oryzae + ltxABC + hetI* strains (Figure S27).

### 2.3. Recombinant Expression of LtxB Does Not Indicate Translational Coupling

**Figure 3:**
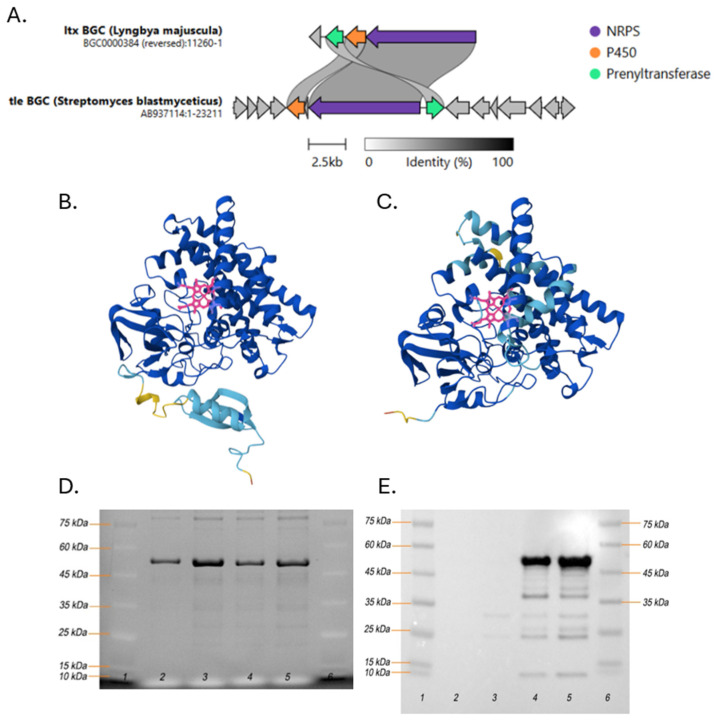
(**A**) Synteny analysis of the *ltx* and *tle* BGCs using clinker; (**B**) AlphaFold model of LtxB with the MbtH domain fusion and the heme cofactor highlighted in pink; (**C**) AlphaFold model of TleB lacking an MbtH domain with heme highlighted in pink; (**D**) SDS-PAGE of recombinant LtxB; (**E**) Western blot of recombinant LtxB using an anti- 6xHis tag antibody. In the gel images, lanes 1 and 6 are the protein ladder; 2 and 3 are the first and second eluates of N-terminal 6xHis tagged LtxB; 4 and 5 are the first and second eluates of C-terminal 6xHis tagged LtxB.

In the homologous *tle* BGC, which produces LTXA as an intermediate during teleocidin B in *Streptomyces blastmyceticus* [22], the P450 enzyme and MbtH protein are encoded by two separate genes (Figure 3A–C). To investigate whether translational features of *ltxB* could contribute to pathway failure in a fungal host, LtxB was heterologously expressed in *Escherichia coli* with either N- or C-terminal 6xHis tags. SDS–PAGE analysis revealed a single protein band at ~53 kDa for both constructs, and Western blot analysis confirmed expression of the C-terminally tagged protein. Peptide sequencing of purified LtxB confirmed that the correct protein had been recombinantly produced; however, the N-terminal His-tagged protein may have lost its His-tag via Ni-NTA affinity column purification (Figures S13 and S14). No evidence for a smaller (~9 kDa) stand-alone MbtH domain was observed. These findings suggest that translational uncoupling of the fused MbtH-P450 architecture is unlikely to explain pathway failure in the fungal host, thereby narrowing the remaining barriers to downstream issues such as codon usage, protein folding, proteolysis, localization, or catalytic compatibility.

## 3. Discussion

Heterologous production of cyanobacterial natural products is notoriously challenging in prokaryotic hosts, yet the reasons are mostly unknown [8,11]. The *ltx* BGC is one of the most well-studied cyanobacterial gene clusters, being heterologously expressed in *E. coli*, *Anabaena* sp., and *Streptomyces* sp. hosts, but has not yet reached industry-relevant yields [9,19,21]. We therefore attempted to heterologously express the *ltx* BGC in *A. oryzae* NSAR1 by cloning individual genes under the control of the strong, inducible *amyB* promoter, essentially overriding prokaryotic mechanisms for operon expression. Although transcripts could be detected for *ltxB, ltxC,* and *hetI/sfp*, neither LTXA, ILV, intermediates, analogues, nor shunt products were detected. The *ltxA* transcript could not reliably be detected, even screening up to 30 transformants, despite the gene being fully integrated into the fungal genome. Challenges with expressing cyanobacterial megasynthetase genes in uncommon hosts appear to be a common phenomenon, as when *ltxA* was heterologously expressed in *Streptomyces coelicolor* A3, a truncated transcript (~2000 bp) was also reported [21]. The reasons proposed for truncation include promoter incompatibility, mRNA secondary structure, and/or rare codons, with the latter two proposals also being relevant to our study. Although it is possible that native splicing machinery in *A. oryzae* was responsible for mRNA truncation in our study, the in silico predictions (Figures S7 and S8) [23,24,25] do not confirm our observations in diagnostic PCR, where an mRNA transcript for segment 2 would still be expected (Figure S5).

The lack of detectable pathway intermediates in *A. oryzae* may also reflect incompatibility of LtxB with the fungal intracellular environment. LtxB is a bifunctional protein comprising an MbtH-like domain fused to a cytochrome P450 monooxygenase. In contrast to eukaryotic P450 enzymes, which are typically anchored to the endoplasmic reticulum via an N-terminal transmembrane helix [26,27], LtxB lacks such an α-helix (Figure 3B). Mislocalization of LtxB in the cytosol may therefore limit access to redox partners required for catalytic activity. In addition, the didomain features of LtxB may present folding challenges in a heterologous host as MbtH-like proteins are often expressed as discrete partners that assist NRPS function [28,29]. Our investigation of LtxB in *E. coli* does not indicate translational coupling in that environment, and we were able to detect full-length *ltxB* transcripts after heterologous expression in *A. oryzae*. Therefore, fusion of a P450 to an MbtH-domain may alter accessibility, folding efficiency, or stability in a eukaryotic system. Future studies could investigate heterologous expression of chimeric P450s with an a-helix anchor, with and without the MbtH-domain, and with cyanobacterial CYP reductases.

Despite these limitations, successful conversion of ILV to LTXA by codon-optimized LtxC demonstrates that downstream tailoring enzymes can remain functional in *A. oryzae* if codon-optimized. This suggests that modular pathway reconstruction offers a viable strategy for engineering cyanobacterial gene clusters in fungal hosts.

## 4. Conclusions

This study demonstrates that full reconstitution of cyanobacterial NRPS pathways in fungal hosts remains challenging, reflecting challenges observed in non-cyanobacterial prokaryotic hosts [7,8,9,10,11]. In addition, the likely incompatibility of LtxB with the fungal intracellular environment further emphasizes the importance of protein localization and domain architecture for pathway functionality. Future work should investigate entirely codon-optimized gene sequences, combined with N-terminal tagged proteins for detection via Western blot, to determine whether *ltxA* can be successfully transcribed and translated in a fungal host. Additionally, further work with a larger selection of cyanobacterial BGCs is needed to establish generalizability.

Importantly, the successful biotransformation of ILV to LTXA by codon-optimized LtxC demonstrates that downstream tailoring enzymes from cyanobacterial pathways can remain functional in *A. oryzae*. These findings support a modular approach to pathway engineering, in which individual enzymes or pathway segments can be selectively reconstructed, and provide opportunities for combinatorial biosynthesis with non-cyanobacterial enzymes. Overall, this work provides practical insight into the limitations and opportunities associated with expressing cyanobacterial biosynthetic pathways in fungal hosts, and highlights key considerations for future efforts to engineer eukaryotic systems for the production and diversification of complex natural products.

## Figures and Tables

**Table 1:** Summary of fermentation optimization attempts. DPY = dextrin peptone yeast extract medium; CZDB = Czapek-Dox broth (+/−supplementation with 2% glucose or 2% starch); *ltxC-*co = codon-optimized *ltxC*.

Strain	Conditions Tested	Metabolites Detected	Outcome
*A. oryzae* (*ltxABC*+*sfp/hetI*)	Multiple media (DPY, CZDB ± glucose/starch), 3–12 days	No NMVT, ILV, LTXA or their analogs	Megasynthetase not produced
*A. oryzae* (*ltxABC+hetI*) + ILV	DPY, 7 days	ILV	No biotransformation despite all genes present and transcribed
*A. oryzae* (*ltxC*-co) + ILV	DPY, 7 days	ILV and LTXA	Successful biotransformation and residual ILV were observed

## Data Availability

The authors declare that the data supporting the findings of this study are available within the paper and its Supplementary Information files. Should any raw data files be needed in another format, they are available from the corresponding author upon reasonable request.

## Supplementary Materials

The following supporting information can be downloaded at: https://www.mdpi.com/article/10.3390/jof12070481/s1, Figure S1: Maps of plasmids used in this study; Figure S2: PCR screening of *A. oryzae* transformants integrating: (A) *ltxA*; (B) *ltxB*; (C) *ltxC*; (D) *hetI*; and (E) *sfp*; Figure S3: *A. oryzae* transformant 18 was confirmed as containing *ltxA*-*C* and *hetI* genes and was subsequently used for RT-PCR, timecourse and media studies ; Figure S4: Transcription analysis of *ltxA-C* and *hetI* in transformants 18–21; Figure S5: PCR strategy to determine where *ltxA* is truncated during transcription; Figure S6: *ltxA* 1st first segment (S1: 1 bp–3303 bp) had positive transcription. *ltxA* 2nd segments (S2: 3303 bp–4290 bp) was not detected; Figure S7: Exon and intron predictions of *ltxA* processed by *Emericella* (*Aspergillus*) sp. using FGENESH; Figure S8: Exon and intron predictions of codon optimized *ltxA* processed by *Emericella* (*Aspergillus*) sp. by FGENESH; Figure S9: Sequence alignment of native *ltxA* sequence compared with FGENESH predicted sequence in *Aspergillus* sp. (top) showing predicted splice sites; sequence alignment of LTXA and codon-optimized LTXA predicted sequence from FGENESH (bottom) showing truncated sequence; Figure S10: Sequence alignment of LtxB, TleB, and Orf5 (MbtH); Figure S11: Models of the MbtH domain from LtxB using different sequence lengths (A–C) and comparison to Orf5 from the tle BGC (D); Figure S12: LTX calibration curve and linear fit curve calculated values; Figure S13: Sequencing results of N-terminal 6xHis-tagged LtxB; Figure S14: Sequencing results of C-terminal 6xHis-tagged LtxB; Figure S15: LCMS chromatogram of LTXA commercial standard (Rt=15.10 min) in positive (black line) and negative (pink line) ion mode; Figure S16: Low-resolution mass spectrum of LTXA commercial standard; Figure S17: LCMS chromatogram of ILV commercial standard (Rt = 8.97 min) in positive (black line) and negative (pink line) ion mode; Figure S18: Low-resolution mass spectrum of ILV commercial standard; Figure S19: UV absorption spectra of commercial standards A) LTXA and B) ILV; Figure S20: LCMS chromatogram of Val-Trp dipeptide commercial standard (Rt =1.90 min) in positive (black line) and negative (pink line) ion mode; Figure S21: Low-resolution mass spectrum of Val-Trp dipeptide commercial standard; Figure S22: LCMS traces of *A. oryzae* NSAR1 cell extracts (CE) and media extracts (ME) after feeding with 25 μg of LTXA. A standard (STD) of LTXA at 12.5 µg/mL is included for reference; Figure S23: HR-LCMS analysis of *A. oryzae* + *ltxABC* + *hetI* cell extracts cultivated in various media; Figure S24; HRMS analysis of *A. oryzae* + *ltxABC* + *hetI* cell extracts cultivated in various media; Figure S25: HR-LCMS analysis of *A. oryzae* + *ltxABC* + *hetI* media extracts cultivated in various media; Figure S26: HRMS analysis of *A. oryzae* + *ltxABC* + *hetI* media extracts cultivated in various media; Figure S27: XICs of ILV and LTXA in the media and cell extracts of ILV fed to *A. oryzae* transformants 18, 21; Table S1: Synthetic genes used in this study with start and stop codons underlined; Table S2: Synthetic gene fragments to reconstruct *ltxA in vivo;* Table S3: Oligonucleotides used in this study; Table S4: Plasmids used in this study; Table S5; Strains used in this study; Table S6: Recipes of media used in this study; Table S7: Recipes of buffers and other chemicals used in this study; Table S8: *In silico* predictions of LTX biosynthetic enzyme localization in a fungal host using different tools; Table S9: Serial dilutions of LTXA and corresponding peak areas used to create the calibration curve; Table S10: Quantification and extraction efficiency of LTXA recovered from feeding experiments using the calibration curve from Figure S12 [30,31,32,33].
